# Association between uric acid and height during growth hormone therapy in children with idiopathic short stature

**DOI:** 10.3389/fendo.2022.1025005

**Published:** 2022-12-02

**Authors:** Jong Seo Yoon, Young Jun Seo, Eun Byul Kwon, Hye Jin Lee, Min Jae Kang, Il Tae Hwang

**Affiliations:** Department of Pediatrics, Hallym University College of Medicine, Chuncheon-si, Gangwon-do, South Korea

**Keywords:** uric acid, children, short stature, growth hormone treatment, height

## Abstract

**Background:**

Serum uric acid (UA) within appropriate levels is reported to be beneficial in patients with idiopathic short stature (ISS). This study aimed to evaluate the association between serum UA levels and height standard deviation scores (SDS) in patients with ISS during growth hormone (GH) therapy.

**Methods:**

A longitudinal study (LG Growth Study) of 182 children (mean age: 7.29±2.60 years) with ISS was performed. All participants were in the prepubertal stage and treated with GH, and the data within a treatment period of 30 months were analyzed.

**Results:**

In the adjusted Pearson’s correlation, UA was significantly correlated with height SDS after controlling for sex, age, and body mass index (BMI) SDS (r=0.22, p=0.007). In the adjusted multiple regression analyses, the height SDS was significantly associated with UA after controlling for sex, age, and BMI SDS (β=0.168, p=0.007). Within the 30-month treatment period, the UA levels significantly increased as the height SDS increased, and the mean UA levels at baseline and 30 months after treatment were 3.90±0.64 mg/dL and 4.71±0.77 mg/dL, respectively (p=0.007).

**Discussion:**

In conclusion, UA is related to height SDS, and GH treatment leads to a significant increase in UA without hyperuricemia. Elevated UA is considered a favorable outcome of GH therapy, and further studies are needed to determine its role as a monitoring tool.

## Introduction

The number of studies on the association of uric acid (UA) on growth is extremely limited compared with that on the association between UA and metabolic problems. The general UA level in children gradually increases from birth to adolescence and that the fastest increase in UA occurs during puberty (1, 2). Sexual differences in serum UA levels begin at puberty and partially result from the direct influence of the muscle mass (3). A previous study investigated the association between UA concentrations and the standard deviation scores (SDS) of insulin-like growth factor 1 (IGF-1) in children and adolescents with idiopathic short stature (ISS); IGF-1 SDS was positively associated with appropriate serum UA concentrations, whereas serum UA levels that were too high or too low were associated with lower IGF-1 SDS values (4). IGF-1 is also well known as an important regulator of muscle mass (5, 6). Obesity is associated with hyperuricemia, and a negative relationship has been reported between hyperuricemia and peak growth hormone (GH) levels in obese children and adolescents (7, 8). Studies have suggested a link between UA and GH status, suggesting that serum UA levels may be related to height during growth. We hypothesized that GH treatment would increase IGF-1 levels and possibly lead to an increase in muscle mass, along with an increase in height, leading to elevated serum UA levels. However, previous studies on the association between serum UA levels and height during the growth of children and adolescents have not yet been reported. Therefore, this study was aimed at analyzing the association between serum UA levels and height in Korean children with ISS and investigating the role of UA on growth by measuring the changes in serum UA levels relative to the height increases following GH treatment.

## Methods

### Study design and participants

We used data from a registry study (LG Growth Study, LGS) designed to investigate the efficacy and long-term safety of GH treatment in short Korean children and adolescents with ISS, growth hormone deficiency (GHD), Turner syndrome, small for gestational age (SGA) without catch-up growth, or chronic renal failure. LGS was registered at ClinicalTrials.gov (identifier: NCT01604395). LGS is a multicenter, non-interventional study, and detailed descriptions of its background have been provided in previous publications introducing cohort characteristics and study protocols (9, 10). Written informed consent was obtained from patients and their parents. This study was conducted in accordance with the Declaration of Helsinki and approved by the Institutional Review Board of the Hallym University Kangdong Sacred Heart Hospital (IRB No. 2021-18-019).

A total of 367 patients with ISS were registered in the LGS between November 2011 and March 2017. The inclusion criteria were:1) height <3rd percentile for sex and age according to the data from the 2017 Korean National Growth Charts for children and adolescents (11); 2) normal GH secretion confirmed by GH levels of ≥10 ng/mL in at least one GH stimulation test; 3) prepubertal children before and during GH treatment (without breast development in girls and testicular volume lower than 4 mL in boys); and 4) treatment period longer than 1 year. ISS patients were treated with GH at a dose of 0.37 mg/kg/wk. The exclusion criteria were: 1) children born SGA; 2) chronic diseases, including chronic kidney disease and malnutrition; 3) brain diseases, such as epilepsy, cerebral palsy, and brain tumor; 4) endocrine diseases, such as GHD, hypothyroidism, precocious puberty, and diabetes mellitus; and 5) chromosomal abnormalities, such as Turner syndrome. A total of 182 patients with ISS met these criteria and were included.

### Measurements

Owing to the nature of the enrolled multicenter and non-interventional studies, all laboratory analyses were performed in accordance with the local standard procedures at each site, without the use of a central laboratory. Other laboratory tests, including glucose, blood urea nitrogen (BUN), creatinine, UA, total protein, albumin, aspartate aminotransferase, alanine aminotransferase, total cholesterol (T-C), triglyceride, IGF-1, and IGF-binding protein 3(IGF-BP3) were performed prior to test drug administration in the GH stimulation test. The pediatric endocrinologists at each center performed the physical examinations to determine the Tanner stage and GH stimulation tests by selecting two of the commonly used methods in clinical practice, such as insulin-induced hypoglycemia, L-dopa, clonidine, and glucagon tests. In all research institutions, height was measured to the nearest 0.1 cm using a Harpenden stadiometer (Holtain Ltd., Crymych, Wales, UK), while body weight was measured to the nearest 0.1 kg using a digital scale. BMI was calculated by dividing the weight by height in meters squared (kg/m^2^). The SDSs for height, weight, and BMI were calculated based on the 2007 Korean National Growth Charts using the LMS method (SDS = [measured value/M]^1/L^/LS; L, lambda for the Box-Cox power for skewness; M, mu for the median; S, sigma for the generalized coefficient of variation) (11). The IGF-1 SDS and IGFBP-3 SDS were calculated based on the reference values for Korean children and adolescents (12). Anthropometric and laboratory measures were obtained at 6-month intervals during the period of GH treatment, and these data were used to analyze the variables related to the changes in UA levels.

### Statistical analysis

Statistical analyses were performed using SAS version 9.4 (SAS Institute, Cary, NC, USA) and the R statistical software (https://www.r-project.org). To compare the statistical significance between groups, Fisher’s exact test or chi-square test was used for categorical variables, while the t-test or Mann-Whitney U test was used for continuous variables. Pearson’s correlation tests were performed at baseline to examine the correlation between UA and other anthropometric and biochemical variables, while partial correlation was used after adjusting for sex, age, and BMI SDS. Multiple linear regression analysis was conducted to investigate the independent association between UA and height SDS, after adjusting for sex, age, and BMI SDS at baseline. The clinical variables were expressed as mean ± standard deviation. Statistical significance was set at *p*<0.05.

## Results

### Baseline clinical characteristics of the participants

The baseline clinical characteristics of the patients are shown in **Table 1**. A total of 182 patients (53.3% were boys) with ISS were included in this study. The mean age of the participants was 7.29 ± 2.60 years. The mean UA levels were 3.95 ± 0.78 mg/dL in boys and 3.79 ± 0.84 mg/dL in girls at baseline, and no significant difference was observed in the UA levels between the two groups.

**Table 1:** Baseline clinical characteristics of the study participants.

Variable	Total (n=182)	Male (n= 97)	Female (n= 85)	*p*
Age, years	7.29 ± 2.60	7.26 ± 2.74	7.32 ± 2.45	0.877
Height, cm	113.68 ± 12.96	112.49 ± 13.34	115.13 ± 12.44	0.214
Height SDS	−2.45 ± 0.66	−2.45 ± 0.45	−2.46 ± 0.85	0.911
Weight, kg	20.87 ± 6.01	21.06 ± 6.65	20.64 ± 5.15	0.671
Weight SDS	−1.93 ± 0.88	−1.86 ± 0.89	−2.01 ± 0.86	0.307
BMI	15.88 ± 1.84	16.16 ± 1.80	15.53 ± 1.83	0.039
BMI SDS	−0.64 ± 1.04	−0.53 ± 1.05	−0.77 ± 1.02	0.182
Glucose, mg/dL	91.41 ± 12.76	92.22 ± 12.14	90.48 ± 13.45	0.361
BUN, mg/dL	12.61 ± 3.02	12.91 ± 2.88	12.28 ± 3.15	0.169
Creatinine, mg/dL	0.44 ± 0.13	0.44 ± 0.13	0.45 ± 0.12	0.853
AST, U/L	30.19 ± 7.45	29.79 ± 6.69	30.65 ± 8.25	0.448
ALT, U/L	14.69 ± 5.94	15.07 ± 6.42	14.26 ± 5.34	0.358
UA, mg/dL	3.88 ± 0.81	3.95 ± 0.78	3.79 ± 0.84	0.188
T-C, mg/dL	168.78 ± 24.40	166.62 ± 26.03	171.19 ± 22.36	0.239
TG, mg/dL	82.72 ± 39.21	78.53 ± 40.83	86.70 ± 38.23	0.522
IGF-1	159.81 ± 85.10	155.83 ± 95.70	164.43 ± 71.27	0.534
IGF-1 SDS	−0.58 ± 0.99	−0.40 ± 1.12	−0.79 ± 0.77	0.013
IGF-BP3	2,924.50 ± 1,372.55	2,758.57 ± 1,206.45	3,129.15 ± 1,549.90	0.275
IGF-BP3 SDS	0.44 ± 2.44	0.41 ± 2.41	0.49 ± 2.52	0.892
Peak GH, ng/mL	18.14 ± 10.08	17.82 ± 8.08	18.56 ± 12.23	0.681

BMI, body mass index; SDS, standard deviation score; BUN, blood urea nitrogen; AST, aspartate aminotransferase; ALT, alanine aminotransferase; T-C, total cholesterol; TG, triglyceride; IGF-1, insulin-like growth factor-1; IGF-BP3, insulin-like growth factor-binding protein 3; GH, growth hormone.

### Correlation between UA and anthropometric and biochemical variables at baseline

Pearson’s correlation analysis was performed to analyze the relationship between UA levels and anthropometric and biochemical variables. As shown in **Table 2**, UA levels had a significantly positive correlation with height SDS in the unadjusted (r=0.18, *p*=0.023) and adjusted (r=0.22, *p*=0.007) correlation analyses. Other variables, including weight, SDS, BUN, total protein, albumin, and T-C, were significantly positively correlated with UA levels in the adjusted correlation analysis (*p*<0.05).

**Table 2:** Unadjusted and adjusted correlation analyses between uric acid and anthropometrical and biochemical variables at baseline.

	Unadjusted model		Adjusted model	
	r	*p*	r	*p*
Sex	-0.10	0.188	–	–
Age	0.00	0.977	–	–
BMI SDS	0.07	0.394	–	–
Height	0.03	0.687	0.15	0.071
Height SDS	0.18	0.023	0.22	0.007
Weight	0.1	0.213	0.24	0.003
Weight SDS	0.17	0.040	0.22	0.009
Glucose	-0.15	0.037	-0.13	0.113
BUN	0.26	<0.001	0.17	0.043
Cr	0.11	0.136	0.16	0.054
Total protein	0.17	0.021	0.31	<0.001
Albumin	0.10	0.163	0.18	0.028
AST	0.10	0.182	0.15	0.072
ALT	0.08	0.270	0.04	0.667
T-C	0.13	0.104	0.20	0.030
TG	0.07	0.667	0.03	0.875
IGF-1 SDS	0.11	0.194	0.16	0.090
IGF-BP3 SDS	-0.02	0.853	0.05	0.739
Peak GH	0.23	0.007	0.11	0.245

BMI, body mass index; SDS, standard deviation score; AST, aspartate aminotransferase; ALT, alanine aminotransferase; T-C, total cholesterol; TG, triglyceride; IGF-1, insulin-like growth factor-1; IGF-BP3, insulin-like growth factor-binding protein 3; GH, growth hormone.

The adjusted model was used after controlling for sex, age, and BMI SDS score.

### Association of height SDS with anthropometric and biochemical variables by multiple linear regression analyses at baseline

Results of the unadjusted and adjusted multiple linear regression analyses of the association between UA and the anthropometric and biochemical variables are shown in **Table 3**. In the unadjusted multiple linear regression analysis, the height SDS was significantly and positively associated with UA levels (β=0.156, *p*=0.023). Height SDS also had a significantly positive association with UA levels in the adjusted multiple linear regression analysis after controlling for sex, age, and BMI SDS (β=0.168, *p*=0.007). The other variable associated with height SDS in the unadjusted and adjusted multiple linear regression analyses was the weight SDS.

**Table 3:** Association of height SDS with anthropometric and biochemical variables using unadjusted and adjusted multiple linear regression analyses at baseline.

	Unadjusted model			Adjusted model		
	β	SE	*p*	β	SE	*p*
Age	−0.022	0.014	0.103	–	–	–
Sex	−0.065	0.074	0.382	–	–	–
BMI SDS	0.033	0.034	0.331	–	–	–
Weight	0.007	0.005	0.154	0.181	0.014	<0.001
Weight SDS	0.300	0.035	<0.001	1.332	0.026	<0.001
Glucose	0.007	0.003	0.331	0.006	0.003	0.333
BUN	0.007	0.013	0.604	0.015	0.013	0.250
Cr	0.336	0.333	0.314	0.519	0.353	0.143
Uric acid	0.156	0.068	0.023	0.168	0.061	0.007
Total protein	0.010	0.091	0.277	0.065	0.088	0.457
Albumin	0.154	0.152	0.312	0.026	0.144	0.859
AST	−0.001	0.006	0.831	−0.001	0.006	0.992
ALT	0.001	0.007	0.827	0.006	0.007	0.437
T-C	−0.001	0.002	0.971	0.003	0.002	0.835
TG	0.115	0.002	0.053	0.002	0.002	0.311
IGF-1 SDS	0.088	0.045	0.052	0.039	0.043	0.365
IGF-BP3 SDS	0.089	0.028	0.002	0.049	0.026	0.061
Peak GH	−0.005	0.005	0.270	−0.008	0.005	0.105

BMI, body mass index; SDS, standard deviation score; AST, aspartate aminotransferase; ALT, alanine aminotransferase; T-C, total cholesterol; TG, triglyceride; IGF-1, insulin-like growth factor-1; IGF-BP3, insulin-like growth factor-binding protein 3; GH, growth hormone.

Multiple analysis was adjusted for age, sex, and BMI SDS.

### Change in anthropometric and biochemical variables during GH treatment

**Figure 1:**
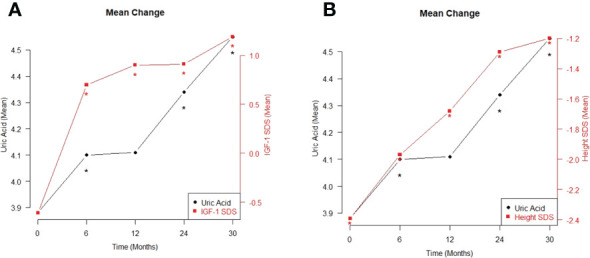
The mean changes in between uric acid and IGF-1 SDS **(A)**, and between uric acid and height SDS **(B)** during the period of growth hormone treatment *p < 0.05.

The different variables monitored within the 30-month GH treatment period are listed in **Table 4** and **Figure 1**. The treatment period was subdivided as follows: before treatment and at 6, 12, 24, and 30 months after treatment. As expected, height, height SDS, IGF-1, and IGF-1 SDS continued to increase from baseline to 6, 12, 24, and 30 months after treatment (*p*<0.001). The UA levels increased significantly during the treatment period, in line with the increasing trend of these factors (*p*<0.001). The BMI also steadily increased significantly during the period of GH treatment (*p*<0.05), but the BMI SDS did not show a statistically significant change.

**Table 4:** Changes in anthropometric and biochemical variables during recombinant human growth hormone treatment.

	Before treatment	After treatment				*p*
		6 months	12 months	24 months	30 months	
Height	114.34 ± 13.74	119.10 ± 13.62^a^	123.44 ± 13.81^b^	129.62 ± 14.79^c^	134.00 ± 15.84^d^	<0.001
Height SDS	−2.52 ± 0.60	−2.11 ± 0.65^a^	−1.78 ± 0.60^b^	−1.44 ± 0.69	−1.20 ± 0.53^d^	<0.001
Weight	21.28 ± 6.71	23.20 ± 7.05^a^	25.65 ± 8.24^b^	30.02 ± 11.00	31.96 ± 11.06^d^	<0.001
Weight SDS	−2.02 ± 0.92	−1.75 ± 0.80^a^	−1.47 ± 0.75^b^	−1.11 ± 0.79	−0.99 ± 0.83^d^	<0.001
BMI	15.88 ± 2.00	15.95 ± 1.83	16.32 ± 2.02	17.16 ± 2.84	17.30 ± 2.72^d^	0.007
BMI SDS	−0.71 ± 1.06	−0.76 ± 0.91	−0.69 ± 0.84	−0.46 ± 0.91	−0.51 ± 1.00	0.628
Uric acid	3.90 ± 0.64	4.04 ± 0.76^a^	4.13 ± 0.87^b^	4.37 ± 0.77^c^	4.71 ± 0.77^d^	<0.001
IGF-1	154.27 ± 74.35	295.87 ± 133.35^a^	341.94 ± 158.81^b^	401.19 ± 185.38^c^	434.52 ± 193.68^d^	<0.001
IGF-1 SDS	−0.70 ± 0.78	0.79 ± 1.29^a^	1.01 ± 1.35^b^	1.02 ± 1.30^c^	1.41 ± 1.64^d^	<0.001
IGF-BP3	2,818.26 ± 1,232.73	3,342.67 ± 1,307.78^a^	3,655.20 ± 1,434.28^b^	3,657.40 ± 1,648.21	3,221.25 ± 1,553.00	0.204
IGF-BP3 SDS	0.28 ± 2.23	1.19 ± 2.34^a^	1.56 ± 2.46^b^	1.58 ± 3.24	0.99 ± 3.47	0.452

Before treatment–12 months after treatment: ^a^p<0.001; 6 months after treatment–12 months after treatment: ^b^p<0.001; 12 months after treatment–24 months after treatment: ^c^p<0.001; 24 months after treatment–30 months after treatment: ^d^p < 0.001.

## Discussion

Our findings identified an independent correlation between height SDS and serum UA levels in children with ISS, as confirmed by multiple regression analysis after adjusting for age, sex, and BMI SDS. More importantly, the serum UA levels significantly increased with elevation in height SDS during the 30-month GH treatment. No hyperuricemia was observed in the GH-treated patients.

The reference range for UA levels in healthy children and adolescents has been reported to have a specific distribution. According to a study of UA reference values for Brazilian children and adolescents, UA slowly increases with age throughout childhood and remains the same in both sexes, and sexual differences in distribution begin at puberty (3). The results of this study also revealed no difference in UA levels between boys and girls before puberty. The relationship between UA and body composition components showed that body fat percentage seemed to have a partial effect in girls, and that the only factor contributing to UA elevation in both sexes was muscle mass. Muscle mass is considered the largest source of purines in the body, and as muscle mass increases, the supply of nucleic acids and purines to the liver increases, resulting in increased UA production (13). Alvim et al. recently reported an association between muscle mass and UA level in children and adolescents. After adjusting for age and fat mass, both sexes with higher muscle mass showed higher UA (3). Previous reports have shown that the body composition of short-stature children has different characteristics than that of normal children. In a case-control study comparing muscle mass between children with short stature and normal controls, fat-free mass, such as protein and bone minerals, was reported to be lower in children with short stature (14).

GH treatment results in improvements in body composition and long-term beneficial effects on muscle and adipose tissues (15, 16). Matusik et al. reported that severe GH deficiency is associated with an increase in adipose tissue and a decrease in muscle mass (17). An increase in muscle volume and strength was observed in adults with GHD treated with GH (16). In children with isolated GHD, GH treatment plays an important role not only in the promotion of linear growth but also in the development of muscles and bones (18). GH treatment in children with SGA, Prader–Willi syndrome, and ISS had a positive effect on muscle mass and strength (19–21). Taken together, this evidence suggests that GH treatment in short-stature children will improve body composition, with long-term beneficial effects on muscle and adipose tissue, and that the increase in muscle mass will eventually lead to an increase in UA levels.

However, little is known regarding the association of GH therapy with UA metabolism. Dixit et al. reported an increase in UA levels after GH treatment in children with short stature aged 4–17 years (22). The serum UA level was 3.4 ± 0.4 mg/dL before GH treatment. The mean elevation of UA was 1.4 ± 1.4 mg/dL after GH treatment and correlated with the mean duration of treatment (2.7 ± 2.1 years). Studies on UA in children with short stature are also rare. Recently, Wang et al. reported an association between UA and IGF-1 SDS in Chinese children and adolescents with ISS (4). They showed a positive correlation between IGF-1 and UA between 2.82 mg/dL and 5.06 mg/dL. However, an inverse correlation was observed between IGF-1 SDS values and UA concentration that were either above or below threshold values; the IGF-1 SDS value decreased in response to changes in UA concentrations that were either less than 2.82 mg/dL or more than 5.06 mg/dL. The present study attempted to reproduce the nonlinear distribution results presented in a previous study using stepwise statistical analysis, but the distribution of serum UA was not characteristic. Moreover, although the increasing trends of IGF-1 SDS and UA levels were similar, no statistical association was observed. Instead, our study showed that UA was significantly associated with height SDS, and that UA increased significantly from 3.90 mg/dL to 4.71 mg/dL after GH treatment. This change in UA level corresponds to the appropriate UA range, as suggested in previous studies. Wilcox et al. reported a mean UA value of 4.1 ± 1.0 mg/dL at age 5–10 years, and Kubota et al. reported that it was 4.2 ± 0.9 mg/dL at age 7– 9 years (23, 24). The 50th percentile of UA for Korean children and adolescents is 5.1 mg/dL, but it cannot be directly compared because it is a value for the distribution at ages 10–18 years (25).

This study, as well as the study by Wang et al. (4), suggests that an increase in UA within an appropriate reference range can have a positive effect on the growth of patients with ISS. This study confirmed that changes caused by GH treatment, including increases in IGF-1 SDS and height SDS, were not different from previously reported results. Based on the results presented above, we predicted that GH treatment in ISS patients would result in an increase in muscle mass along with an increase in height SDS, which would eventually lead to an increase in UA. The results of this study suggest that UA may serve as a monitoring tool for GH treatment.

A major limitation of this study was that data such as dual-energy X-ray absorptiometry related to body composition measurements were not available. Therefore, we could not confirm an association between UA elevation and changes in body composition. Another limitation is that UA can be affected by food intake; however, it was not possible to analyze this because of the nature of this study. Nevertheless, this study had several strengths. In this study, to minimize the factors affecting UA, we included children with no underlying medical conditions such as chronic disease, endocrine disease, brain disease, or chromosomal abnormalities. Furthermore, considering that the characteristics of UA are affected by puberty, only prepubertal patients were included; thus, it is considered a meaningful result compared with other studies that included pubertal patients. The characteristics of multicenter long-term prospective cohort studies may also lend greater significance to the statistical results of this study.

In conclusion, serum UA levels are associated with height SDS. The result of a significant elevation in serum UA with an increase in height SDS after GH treatment was an interesting feature found during GH treatment. Further studies are needed to determine whether changes in serum UA levels caused by GH treatment are associated with changes in height and body composition, and to determine the role of UA in monitoring GH treatment.

## Data availability statement

The original contributions presented in the study are included in the article/supplementary material. Further inquiries can be directed to the corresponding author.

## Ethics statement

The studies involving human participants were reviewed and approved by Institutional Review Board of Hallym University Kangdong Sacred Heart Hospital. Written informed consent to participate in this study was provided by the participants’ legal guardian/next of kin.

## Author contributions

JSY contributed to the data research and analyses and drafted the initial manuscript; YJS, EBK, and HJL designed the study and interpreted the findings; MJK critically reviewed the manuscript and contributed to the conception of the study; and ITH designed and conceptualized the study and critically reviewed the manuscript. All authors contributed to the article and approved the submitted version.

## Acknowledgements

We thank all the physicians who provided the data of patients included in the “LG Growth Study”; the authors would also like to thank LG Chem, Ltd., for assisting with the statistical analysis.

## Conflict of interest

The authors declare that the research was conducted in the absence of any commercial or financial relationships that could be construed as a potential conflict of interest.

## Publisher’s note

All claims expressed in this article are solely those of the authors and do not necessarily represent those of their affiliated organizations, or those of the publisher, the editors and the reviewers. Any product that may be evaluated in this article, or claim that may be made by its manufacturer, is not guaranteed or endorsed by the publisher.
